# A PILOT TEST OF A NOVEL MEDICAL EDUCATION DEVICE FOR LVAD PATIENTS

**DOI:** 10.1093/geroni/igae098.3402

**Published:** 2024-12-31

**Authors:** Jennifer Crittenden, Hannah Longley, Asheesh Lanba, Amarpreet Kohli, Nihar Kumthekar, Jacob Inger, Killian Hadsell

**Affiliations:** University of Maine, Orono, Maine, United States; University of Maine, Bangor, Maine, United States; University of Southern Maine, Portland, Maine, United States; University of Southern Maine, Portland, Maine, United States; University of Southern Maine, Portland, Maine, United States; University of Southern Maine, Portland, Maine, United States; University of Southern Maine, Portland, Maine, United States

## Abstract

Medical assist devices, like the Left Ventricle Assist Device (LVAD), are increasingly used by individuals 65 and older. This project entails a pre-clinical trial feasibility study of a novel, non-invasive, mock LVAD device (the XVAD). Phase I testing consisted of adults under the age of 65 (N = 8, M = 28.5 years) and Phase II expanded study enrollment into the 65-90 age group (N = 4, M = 71 years). All participants wore the device for three to five days and experienced random device alarms similar to those provided by an LVAD device. Each alarm and participant response time was recorded by the XVAD device. Real time response data was supplemented with survey data, qualitative diary data, and feedback gathered via a debrief interview. Qualitative findings underscore the following: Importance of accessible device design for older adults, the influence of participant activity on alarm response times, the need for clarity related to alarm responses, and the impact of XVAD usage on daily activities. Findings indicate that feedback across phases, though they engaged different age groups, yielded convergence around accessibility concerns. Examination of response time device data indicates that while older and younger participants demonstrated different reaction times at the start of the trial, the groups demonstrated equivalent reaction times to device alarms at the end of each trial indicating the potential for effective patient training over time. Findings will be used to inform device development and can inform similar mock device trials that use a multi-generational phased testing approach.

